# Supplementation with Silk Amino Acids improves physiological parameters defining stamina in elite fin-swimmers

**DOI:** 10.1186/s12970-014-0057-4

**Published:** 2014-11-30

**Authors:** Igor Z Zubrzycki, Zbigniew Ossowski, Stanislaw Przybylski, Magdalena Wiacek, Anna Clarke, Bartosz Trabka

**Affiliations:** Jędrzej Śniadecki Academy of Physical Education and Sport ul, Kazimierza Górskiego 1, Gdańsk, 80-336 Poland; Department of Microbiology and Biochemistry, University of Fort Hare, P/B X1314, Alice, 5700 South Africa; School of Health and Applied Sciences, Polytechnic of Namibia, Private Bag 13388, 13 Storch Street, Windhoek, Namibia

**Keywords:** Silk amino acids, Stamina, Testosterone, Human

## Abstract

**Background:**

Previous animal study has shown that supplementation with silk amino acid hydrolysate (SAA) increases stamina in mice. The presented study was the first formal evaluation of the influence of SAA supplementation on parameters defining physiological fitness level in humans.

**Methods:**

It was a randomized controlled trial with a parallel-group design on elite male fin-swimmers. The experimental group was supplemented with 500 mg of SAA per kg of body mass, dissolved in 250 ml of a Carborade Drink®; the control group with Carborade Drink® alone; 3 times a day, 30 minutes prior to the training session.

**Results:**

Changes discerned in the experimental group were more pronounced than those observed in the control group. For example, the change in the serum lactic acid concentration observed in the experimental group was sevenfold less than in the control group [21.8 vs. -3.7 L% for the control and experimental groups, respectively]. An analysis of a lactate profile as a function of a maximal swimming velocity exposed a statistically significant positive shift in the swimming velocity of 0.05 m/s, at the lactate concentration of 4 mmol/L in the experimental group. There was also a positive, although statistically insignificant, increase of 2.6 L% in serum testosterone levels in the experimental group.

**Conclusions:**

This study showed that a 12-day SAA supplementation combined with an extensive and rigorous training schedule was sufficient to increase an aerobic stamina. However, this phenomenon was associated with an augmented level of muscular damage (an increased level of creatine phosphokinase in the experimental group).

## Introduction

A shorter running time, a longer jump, or a longer distance run during a specific time, are results of not only a rigorous training regime but also of an increase of understanding of human physiology combined with modern nutritional techniques.

Recently, a hydrolysate of silk amino acids (SAA) from silk of silkworms (*Bombyx mori*) comprising of peptides of a length of 18 to 19 amino acids, has become very popular among people performing leisure and competition sports.

According to a variety of findings employing animal models, SAA exerts a vista of physiological actions including, among others, anti-diabetic [1], anti-oxidant [2], and anti-tumor [3] properties. One study had even shown that SAA may also influence levels of blood triglycerides [4].

Although some study indicated that branched-chain amino acids (BCAA) supplementation (300 mg/kg of body mas/day) may enhance exercise capacity [5] recent studies on an animal model, performed by South Korean scientists, showed that high doses of SAA, i.e., 800 mg/kg of body-mass [6] or 500 mg/kg [7] also enhance stamina. The latter also shows a clear augmentation of testosterone production.

Although, as stated above, SAA and BCAA evoke similar physiological response, i.e., enhancement of exercise capacity, the obvious differences in their amino acid compositions – BCAA comprises of Leu, Ile, and Val whereas SAA: Ala, Gly, Ser, Val, and Thr - enforces, most probably, different modes of their metabolic action. It is known that BCAAs are mainly oxidized in skeletal muscle and that exercises promote oxidation of BCAA [8]. The mode of action of SAA is currently not established, although there are some indications that its oxidation will occur mainly in liver and the main amino acid, playing the paramount role in its physiological function, is L-alanine -through its conversion to beta-alanine [9].

Since SAA is already on shelves of many sport nutrition shops, we undertook the task of verifying its stamina-enhancing viability on a human model. The presented study was a randomized controlled trial with a parallel-group design on male competitive fin-swimmers.

## Methods

### Ethics

All procedures used in this study were in accordance with the ethical standards of the regional medical chamber. A written consent was obtained in writing from participants and/or guardians of participants under the age of 18 years.

### Study subjects

We conducted a randomized controlled trial with a parallel-group design of competitive male fin-swimmers. All participants were recruited from the national fin-swimmer team.

Both samples’ size was eight (i.e., the control and the experimental sample), and the age means and standard deviations were 17.6 ± 3.78 and 20.71 ± 4.82, respectively.

### Training program

The training camp, during which the experiment was performed, was a microcycle lasting 12 days and comprising three training units daily: (1) 7:00–9:30 am swimming, (2) 11:00–1:00 pm walking and/or running, every other day stretching, and (3) 3:00–5:00 pm swimming.

Within this microcycle, each of the competitors performed 30 training units constituting of total 65training hours. Each of the subjects swam, on average, 7 km per day.

### Supplementation

All the required supplements were bought directly from a distributor (Fitness Authority®, http://www.fanutrition.pl/en) without an indication of the purpose of the purchase. According to the producer, the supplement contained 85.95 g of SAA (alanine, glycine, serine, valine, and threonine) per 100 g of dry mass. A required daily amount of SAA, comprising total of 500 mg SAA per kg of body mass, was dissolved in 250 ml of a Carborade Drink® containing 6.3 g of carbohydrates, 0.046 g of sodium, 2.4 mg of niacin, 1.8 mg Vitamin E, 0.9 g pantothenic acid, 0.21 g Vitamin B6, and 7.5 μg of Biotin per 750 ml. A respective nutrition, i.e., a mixture of SAA and Carborade Drink®, and Carborade Drink® alone was served through a straw in lidded cups to both the experimental and the control groups three times a day30 minutes before each training session [10]. It has to be stressed that all drinks were prepared by a person who did not have an access to competitors.

During the study, we also monitored the total calories, proteins, carbohydrates, and lipids consumed by the control and the experimental groups, stratified into breakfast, lunch, and supper.

### Data acquisition

A fasting blood draw was completed to measure the following parameters: (1) creatine phosphokinase (CPK), (2) aspartate aminotransferase (AS), (3) alanine transaminase (ALT), (4) lactate dehydrogenase (LDH), (5) serum creatinine (SCr), (6) lactic acid (LH), (7) serum glucose (Gluc), (8) total blood protein (TBP), (9) corticosterone (C), (10) testosterone (T), and (11) blood urea nitrogen (BUN). All laboratory analyses were performed using the ARCHITECT ci8200 Integrated System, Abbott Diagnostic.

### Lactate profiling protocol

The incremental 7 × 200 m step test was used to provide objective information on the aerobic fitness of a swimmer. All testing was conducted in a 50 m pool. Individualized target times, based on the personal best time for each swimmer, were calculated before each test. The final swim was set to be for maximum effort. The time for each 100 m split and the total 200 m was recorded manually. After completion of each 200 m, heart rate was measured with a Polar Sports Tester PE (Polar Electro Oy, Kempele, Finland) and a 25 mL capillary blood sample was taken from the earlobe or finger tip and analyzed for lactate concentration using the Accusport Blood Lactate Meter (Boehringer Mannheim, Germany). Lactate tolerance was assessed graphically on a plot of swimming velocity (swimming pace, m/s over the distance of 200 m) versus lactate concentration.

### Statistical analysis

Changes induced by the training period in experimental and control groups were examined using the Wilcoxon signed-rank test. Differences between the groups were evaluated using the Wilcoxon rank sum test using the P-value of 0.05 as the statistical threshold.

Relative changes induced by the experiment were measured using the “natural” relative difference, employing the natural logarithm, which was denoted as log percent (L%) [11]: L% =100 * ln (after/before).

## Results

The basic statistics of the baseline and the experimental period are shown in Table 1.

**Table 1:** **Differences in physiological parameters at baseline and after 12-days training program in SAA supplemented group and a control group**

**Variable**	**Cont (0)**	**Ex (0)**	**Cont (12)**	**Ex (12)**	**L% Cont**	**L% Ex**
LA	0.82 (0.76,0.9)	1.1 (0.98,1.1)	1.02 (0.92,1.15)	1.06 (0.905,1.2)	21.8	−3.7
G	94 (92,101)	90 (90,93)	80 (74,81)	85 (80,87)	−16.1	−5.7
CPK	195 (168,215)	168 (113,368)	290 (288,297)	413 (331,568.5)	39.7	89.9
LDH	310 (272,314)	298 (266,310)	389 (303,391)	339 (305.5,398.5)	22.7	12.9
AST	18 (17,18)	21 (15,26)	29 (21,29)	27 (25,32.5)	47.7	25.1
TBP	6.7 (6.5,6.9)	6.8 (6.8,7)	6.5 (6.4,6.8)	6.6 (6.5,6.7)	−3.0	−3.0
ALT	17 (15,20)	22 (15,29)	25.5 (22.75,28.75)	22 (20.5,27)	40.6	0
SCr	1.08 (1.01,1.08)	1.19 (1.16,1.27)	0.93 (0.91,0.99)	1.26 (1.165,1.32)	−15.0	5.7
BUN	28 (26,29)	32 (30,32)	24 (24,27)	27 (26,30)	−15.4	−17.0
C	39.7 (30.3,50.3)	52.3 (40.8,53)	38.9 (33.8,39.2)	58.3 (52.2,64.55)	−2.0	10.9
T	23.92 (21.01,28.07)	20.57 (19.43,26.64)	22.51 (21.49,22.8)	21.12 (15.73,24.69)	−6.1	2.6

Ex = experimental group; Cont = control group; LA = Lactic Acid (mmol/L); G = blood glucose (mg/dL); CPK = creatine kinase (U/L); LDH = lactate dehydrogenase (U/L); AST = aspartate aminotransferase (U/L); TBP = Total Blood Protein (g/dL); ALT = alanine transaminase (U/L); SCr = Serum creatinine (mg/dL); BUN = blood urea nitrogen (mg/dL), C = corticosterone (nmol/L); T = Testosterone (nmol/L). The values are presented as a median and an inter-quartile range.

An analysis of changes induced by SAA supplementation in experimental and control groups revealed the lack of statistically significant changes. However, the majority of changes observed in the experimental group are more pronounced than those observed in the control group.

A statistical comparison of the total calories, proteins, carbohydrates, and lipids consumed during the breakfast, lunch, and supper, revealed the lack of significant differences between the control and experimental group.

A concentration of serum lactic acid in the control group elevated (21.8 L%), and decreased (−3.7 L%) in experiment group.

Corticosterone concentration decreased in the control group (−2.0 %L) while in the experimental group, it increased (10.9 L%). Analogous, although not so pronounced, a decrease of −6.1 L% and an increase of 2.6 L% of testosterone levels in the control and experimental groups was observed. It has to be noted that in the previous study an increase of only 1.3 L% in mean testosterone levels after 44 days of exercise program was observed [7].

An increase of 47.7 L% in the control group versus the lack of any changes in the experimental group was observed for ALT. AST concentration increased in both groups, and an increase in the control group was more pronounced than in the experimental group.

There was also a substantial increase in serum creatine kinase levels in both control and experimental groups.

**Figure 1:**
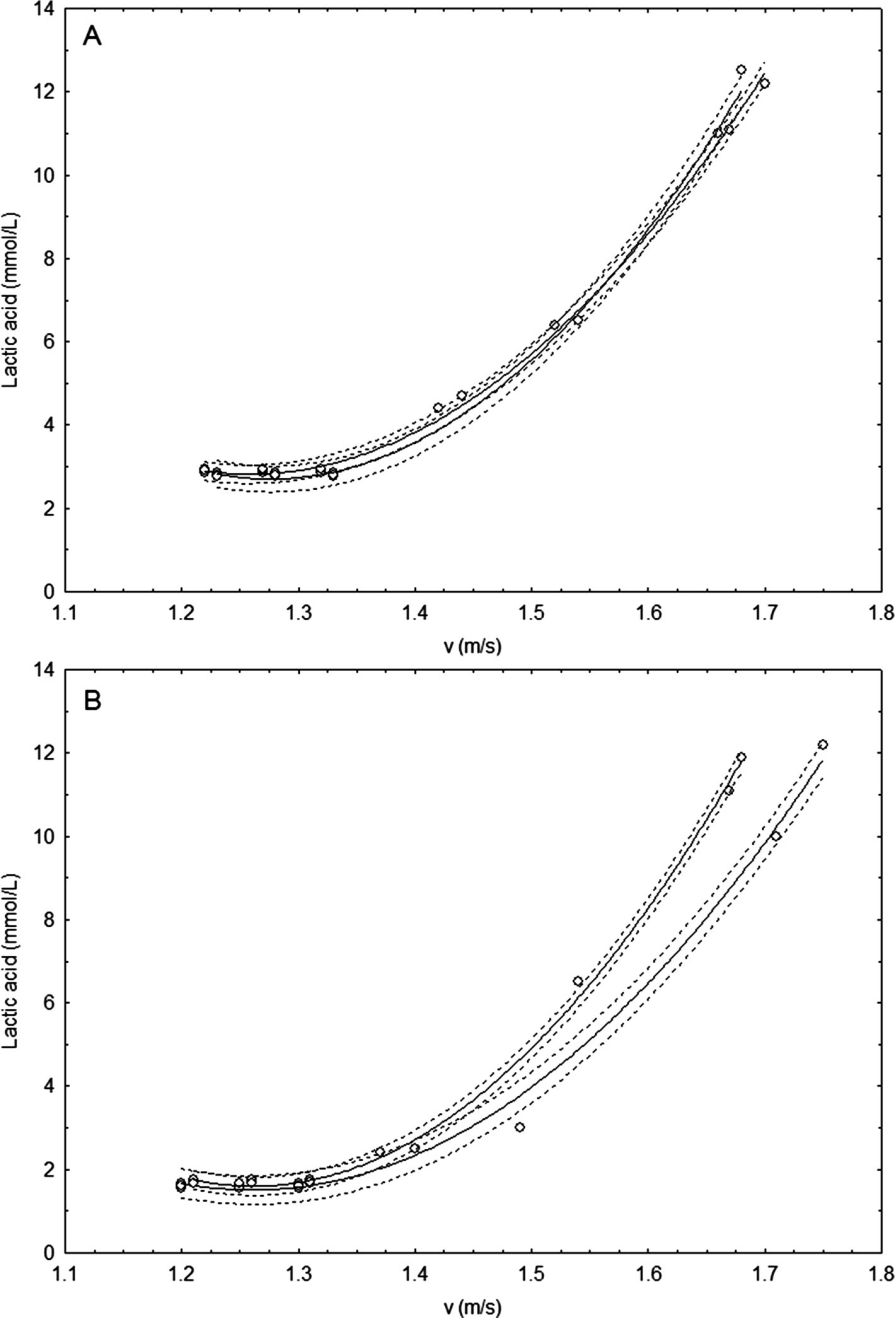
**Blood Lactic Acid (mmol/L) concentration in the control group (A) and the experimental group (B) of elite male fin-swimmers athletes before and after 12 days training microcycle.**

An analysis of a lactate profile, Figure 1A-B, as a function of a maximal swimming velocity, exposed a statistically significant positive shift of swimming velocity, at the reference of the lactate concentration of 4 mmol/L, between the experimental group and the control group.

## Conclusions

A search for novel supplements, other than anabolic steroids, lead to founding of a well-prospering industry, providing amateur and professional sportsmen with a vista of nutrition increasing muscular gain and stamina.

Among them, SAA has become very popular due to their supposed positive influence on physical stamina. Unfortunately, the popularity of this supplement is rendered rather by claims of a manufacturer and not lucid scientific reports on human models. To address this shortcomings we performed this study, which is the first ever attempt to analyze an impact of SAA supplementation on physical stamina in humans.

Taking into account the current literature on translation of drugs dose from animal to human [12], we primarily contemplated the use of ~41 mg of SAA per kg of body mass/day, not 500 mg/kg/day as presented in this study. However, we did not consider SAA as drugs, *per se*, but only as a nutritional supplement. Additionally a limited duration of an experiment i.e., 12-day training regime, and demands of team coaches inclined us to employ a very high dose of SAA. i.e., 500 mg/kg body mass/day.

In this study, performed on a group of competitive male swimmers, we confirmed the earlier finding, that indicated SAA induced increase in physical stamina; *vide* an increase of a maximal swimming velocity at the lactate threshold of 4 mmol/L [13,14] in the experimental group.

Although the previous study showed a positive influence of SAA supplementation on the elevation of testosterone levels on the 44^th^ day program, in which mice were administered to a weight-loaded (5% of body weight) forced swimming, this study, i.e., a 12-day SAA supplementation program combined with an extensive and rigorous training program, resulted only in an slight increase in serum testosterone levels.

There is a significant discrepancy regarding the levels of creatine phosphokinase (CPK), a marker of muscle damage, between this report and the previous one [7]. Opposite to the animal model study, this analysis shows a perspicacious increase in CPK levels in the experimental group as compared to the control group; a change of 36.7 L% in the former versus 89.9 L% in the latter. This observation allows us to conjecture that SAA supplementation induces a higher stamina, but it does not prevent a muscular damage caused by an extensive training effort. However, an analysis of the current literature indicated that supplementation with branched-chain amino acids and taurine-the supplement diminishing or preventing muscular damage caused by extensive training-may be the right direction for utilizing the whole potential of SAA supplementation [15].

The weak point of this study is small sample size, which may be responsible for the lack of more pronounced changes induced by SAA supplementation. Nevertheless, a comparison of our study’s data with those performed on an animal model [7], where the study sample comprised only 10 animals, supported the practical viability of the obtained results.
